# Just-In-Time Adaptive Intervention to Sit Less and Move More in People With Type 2 Diabetes: Protocol for a Microrandomized Trial

**DOI:** 10.2196/41502

**Published:** 2023-09-06

**Authors:** Reza Daryabeygi-Khotbehsara, David W Dunstan, Sheikh Mohammed Shariful Islam, Yuxin Zhang, Mohamed Abdelrazek, Ralph Maddison

**Affiliations:** 1 Institute for Physical Activity and Nutrition (IPAN) Deakin University Geelong Australia; 2 Baker-Deakin Department of Lifestyle and Diabetes Deakin University Melbourne Australia; 3 School of Information Technology Deakin University Geelong Australia

**Keywords:** diabetes, just-in-time adaptive intervention, JITAI, micro-randomized trial, MRT, physical activity, sedentary behavior

## Abstract

**Background:**

Reducing sedentary behavior and increasing physical activity in people with type 2 diabetes (T2D) are associated with various positive health benefits. Just-in-time adaptive interventions offer the potential to target both of these behaviors through more contextually aware, tailored, and personalized support. We have developed a just-in-time adaptive intervention to promote sitting less and moving more in people with T2D.

**Objective:**

This paper presents the study protocol for a microrandomized trial to investigate whether motivational messages are effective in reducing time spent sitting in people with T2D and to determine what behavior change techniques are effective and in which context (eg, location, etc).

**Methods:**

We will use a 6-week microrandomized trial design. A total of 22 adults with T2D will be recruited. The intervention aims to reduce sitting time and increase time spent standing and walking and comprises a mobile app (iMove), a bespoke activity sensor called Sedentary Behavior Detector (SORD), a messaging system, and a secured database. Depending on the randomization sequence, participants will potentially receive motivational messages 5 times a day.

**Results:**

Recruitment was initiated in October 2022. As of now, 6 participants (2 female and 4 male) have consented and enrolled in the study. Their baseline measurements have been completed, and they have started using iMove. The mean age of 6 participants is 56.8 years, and they were diagnosed with T2D for 9.4 years on average.

**Conclusions:**

This study will inform the optimization of digital behavior change interventions to support people with T2D Sit Less and Move More to increase daily physical activity. This study will generate new evidence about the immediate effectiveness of sedentary behavior interventions, their active ingredients, and associated factors.

**Trial Registration:**

Australian New Zealand Clinical Trial Registry ACTRN12622000426785; https://anzctr.org.au/Trial/Registration/TrialReview.aspx?id=383664

**International Registered Report Identifier (IRRID):**

DERR1-10.2196/41502

## Introduction

### Overview

People with type 2 diabetes (T2D) have higher levels of sedentary behavior (SB) and lower levels of moderate to vigorous intensity physical activity (PA) compared with those without T2D [1]. Device-based measurement of SB has shown that people with T2D spend >64% of their daily waking time sedentary [1], which has subsequently sparked calls for targeted behavior change programs to reduce SB in this population, in addition to the promotion of regular PA [2].

SB has become a distinct target for individuals who are susceptible to or have been diagnosed with T2D [3]. It is suggested that people with T2D break up their sitting time by light PA, which can serve as a means for gradually adopting a more active lifestyle [4]. Reducing sitting time by either standing or light ambulation has been shown to improve glucose homeostasis among people at risk of or diagnosed with T2D [3].

Ubiquitous mobile health (mHealth) technologies such as smartphones and wearable sensors offer considerable potential to improve access to and delivery of behavior change support for reducing SB and promoting PA [5,6]. Progress in mHealth technology has enabled the design of just-in-time adaptive interventions (JITAIs), which aim to deliver behavior change support in real or near time, matched to when users need or want the support [7,8]. JITAIs harness information captured through mHealth technologies (smartphone sensors, GPS, and internet access) to determine which behavior change support component should be delivered and tailor it to an individual’s changing context (where) or status (when) [9]. The delivery of more contextually aware intervention support at more salient times to end users is hypothesized to enhance behavior change compared with other delivery approaches (eg, face-to-face and websites) [9].

It has been argued that conventional experimental designs are not sufficient to support the development of JITAIs because they do not enable researchers to determine empirically when a particular intervention component should be delivered and whether a JITAI that was delivered exerted the intended effect [10]. Traditional randomized controlled trial designs evaluate the overall effect of an intervention on a specific behavior or health outcome, not specific components of that intervention. Moreover, randomized controlled trials do not provide information on the particular time when the intervention had an effect and the moderators that affected the behavior change [11].

Other study designs, such as factorial designs, enable researchers to determine the effects of each intervention component, the interactions between components, and the dosing of the intervention. However, they are unable to delineate the time when the intervention was most receptive and what moderators influenced the intervention [11]. To address these limitations, Klasnja et al [11] proposed the microrandomized trial (MRT), which, they argued, could supplement the use of behavioral theory to guide the development of JITAIs. The MRT design provides data on the effects of specific intervention components, changes in effects over time, and potential moderators, including contextual and psychological factors [11]. Due to the dynamic nature of SB and PA that change throughout the day and in various contexts, adaptive designs such as MRT align well with interventions targeting SB and PA behavior change [12]. The MRT design has been suggested as necessary to leverage the potential of smartphones as a tool to improve PA and reduce SB [13]. MRT enables assessing the immediate effects of antisedentary and walking interventions [14] and therefore contributes to the optimization of SB and PA interventions.

Microrandomization refers to randomly allocating an intervention option at relevant times when a person would be most responsive to an intervention (based on theory, the person’s past behavior, and the current context), thereby enhancing the effectiveness of the intervention [11]. MRTs explain the causal relationship between each randomized intervention component (eg, Sit Less or Move More notifications) and the proximal effects (ie, what happens in a limited time window, eg, within 1 hour, following the randomized intervention component) [11]. The distal outcome results from an aggregation of proximal effects obtained through repeated exposure to intervention components over time [11]. For example, a distal outcome would be people achieving recommended levels of PA and longer-term clinical outcomes such as improved glycemic control or improved fasting or postprandial glucose [15].

### Aims

The primary aim of this study is to assess the effectiveness of theory-based motivational messages for reducing proximal sitting time and increasing proximal standing or walking time (ie, nonsitting time). The secondary aims involve (1) assessing what type of behavioral components (ie, behavior change techniques) are most effective in reducing sitting time and increasing standing and walking time, measured proximally, and (2) determining relevant contextual moderators for reducing sitting time and increasing standing and walking time.

## Methods

### Design

**Figure 1:**
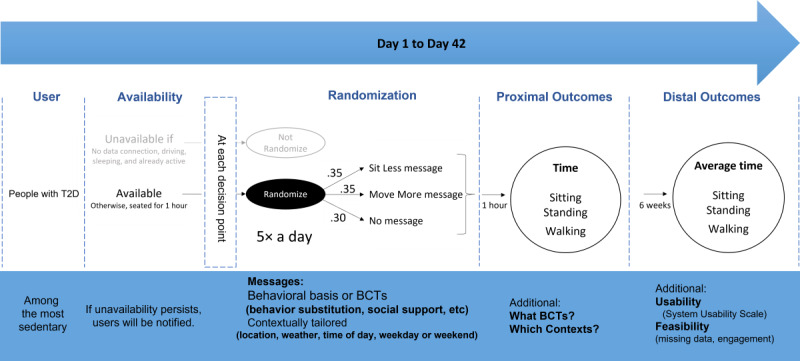
A summary of the microrandomized trial (MRT) design. Target group, availability conditions, study duration and arms, randomization probability, and outcomes are presented. BCT: behavior change technique; T2D: type 2 diabetes.

A 6-week MRT will be conducted (see Figure 1). Using this design, participants will be randomly assigned an intervention option (Sit Less message, Move More message, or no intervention) at each relevant decision point.

### Participants and Recruitment

#### Overview

Participants will be recruited digitally through social media advertisements (eg, Facebook and Instagram). Moreover, posters will be placed on noticeboards of general practitioner and specialist clinics, and emails will be sent to potential participants who have indicated their willingness to participate in research studies. The plain “language statement and consent form” will be sent to eligible participants who express their interest in participating before any data collection.

#### Sample Size

A total of 22 participants are estimated to detect a small proximal treatment effect size of 0.13 for increasing nonsitting time with 80% power at the level of 5% statistical significance. This was based on the assumptions of 5 decision points per day, a constant randomization probability of 0.7, and an expected participant availability of 70% [16].

### Eligibility Criteria

Eligible participants will be adults with T2D, aged between 35 and 65 years, who own an Android smartphone (version 7.0 or higher) and currently interact or engage with apps, have no limitations (including physical problems and medical complications) to engage in low- and moderate-intensity PA, are able to communicate in English, and can provide informed consent.

Volunteers who use other activity trackers and mobile apps that target PA, highly active people, those diagnosed with T2D less than 3 months ago, and people with psychiatric problems, attention, or memory issues will be excluded. We assume highly active people as those who meet World Health Organization (WHO) recommendation of 10,000 steps per day or 150 minutes of moderate to vigorous PA per week. The International Physical Activity Questionnaire—Short Form will be used to assess activity levels [17]. A 1-week run-in period will assess nonadherence with the activity tracker. Individuals with more than 50% incomplete data will be excluded.

### Intervention Platform

**Figure 2:**
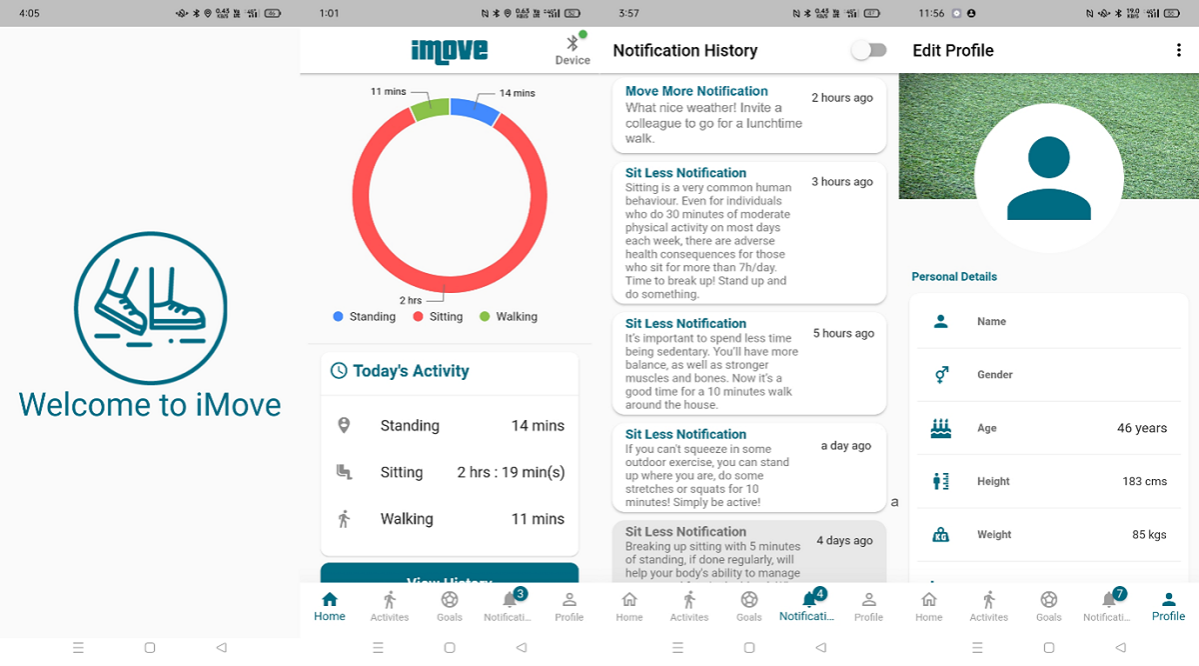
iMove app images.

**Figure 3:**
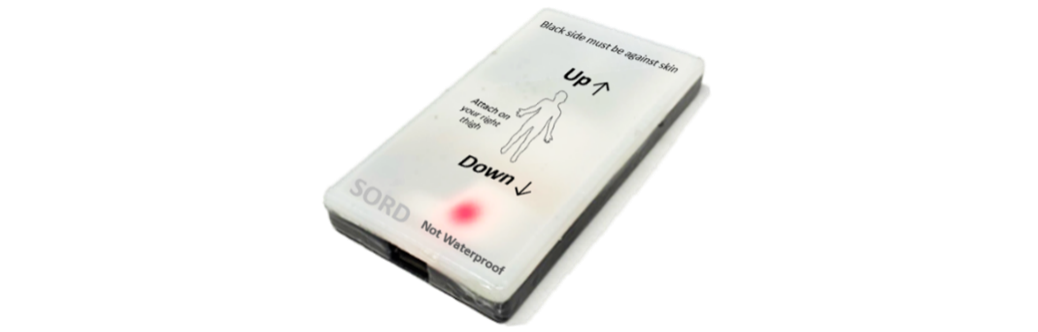
Sedentary Behavior Detector (SORD) device.

The intervention comprises a bespoke mobile app (iMove app) [18] and a Bluetooth-enabled wearable sensor (SORD) developed and validated by our research group [19]. iMove is a JITAI that aims to support people to decrease sedentary time (Sit Less) and increase PA (Move More) [20]. It consists of a home tab that illustrates sitting, standing, and walking activities, an activity tab to select activities manually in situations where technical issues exist with the app, a goal setting tab to set sitting and walking goals, a notification history tab to compile previous notification messages, and a profile tab (see Figures 2 and 3). SORD is a bespoke thigh-worn device that connects to the iMove app and measures sitting, standing, lying, and PA (eg, walking) [19].

### Intervention

#### Overview

Through the iMove app, participants will receive a series of Sit Less and Move More intervention messages (notifications) to serve as cues to action [21] and increase readiness to engage in activity. A pop-up message will appear on the phone screen with a sound effect when a notification is received. There will be 2 intervention options, which include Sit Less (sedentary interruption and transition to standing) and Move More (walking) for 1.5-3 minutes, approximately equivalent to 500-1000 steps, and a control option (no messages). Participants can switch off the push notification for a certain amount of time on any given day.

A list of messages is provided in Multimedia Appendix 1.

Notifications are adapted to 4 different context dimensions, including time of day, day of the week (weekend vs weekdays), location (home vs workplace), and weather conditions (good or suitable for PA, rainy and cold or not suitable for PA). Notifications are delivered on 5 occasions (decision points) throughout the day and include morning, lunchtime, afternoon 1, afternoon 2, and evening (details are presented in the “Decision Points” section below).

At each decision point, participants are randomized only if they are available (ie, they are not driving, not sleeping, not active already, and are connected to the internet). The following probabilities are used for randomization: 70% in the intervention (35% SB, 35% PA) and 30% as active control at each particular decision point. GPS will assist with the recognition of driving if the user is on the move. A preset time (9 PM to 8 AM) will be considered for rest and sleeping. In addition, both driving and sleeping can be manually modified by users.

#### Decision Points

A total of 5 decision points, from 8 AM to 8:30 PM, will be selected as shown in Table 1.

**Table 1:** Occasions (decision points) when notifications are delivered.

Decision point	Time
Morning	8 AM to 10:30 AM
Lunchtime	10:30 AM to 1 PM
Afternoon 1	1 PM to 3:30 PM
Afternoon 2	3:30 PM to 6 PM
Evening	6 PM to 8:30 PM

#### Intervention Messages

Notifications were developed by the research team and incorporate effective behavior change techniques to Sit Less and Move More. Behavior change techniques including behavior substitution [22], social support [23], problem-solving or barrier identification [23], instruction on how to perform the behavior [22,24], providing information on the consequences of PA specific to the individual [23], prompts or cues [24], prompting generalization of a target behavior [23], goal setting [23], self-monitoring [22], and feedback on behavior [22] were used to create messages. Table 2 presents examples of intervention messages and their associated behavior change techniques.

**Table 2:** Examples of Sit Less and Move More messages and their respective behavior change techniques.

Message type and behavior change technique			Example message
**Sit Less**			
	Behavior substitution	You have been sitting for about 1 hour this morning. Why not stand up and do some stretches for 2-3 minutes.	
	Social support	Ask your partner to do some house chores with you (e.g., sweeping, mopping, vacuuming, etc.). It is more fun when you do this together while talking as well!	
	Problem-solving or barrier identification	You might not notice your long periods of sitting time, but it adds up to a lot of hours (e.g. we may sit during meals, travelling to/from work, in front of the computer, watching TV,…). Now it’s the right time to take a break and stand up and do some stretches or warm-up exercise.	
	Instructions on how to perform the behavior	Time to stand up: you may want to grab a drink of water, talk on the phone while walking around or go and talk to your colleagues instead of emailing them.	
	Providing information on the consequences of physical activity (PA) specific to the individual	It’s important to spend less time being sedentary. You’ll have more balance, as well as stronger muscles and bones. Now it’s a good time for a 10 minutes walk around the house.	
	Prompts or cues	Why not go take one of your favorite books and enjoy reading! This way you break up your sitting.	
	Prompting generalization of a target behavior	It looks like you are doing well reducing your sitting time during the mornings. Try to break your sitting time in the evenings as well. It’s the time!	
	Goal setting	Your daily sedentary behaviour goal is *[<VALUE hours]*. While working, you can stand up and do some stretches. This helps you achieve your daily goal.	
	Self-monitoring	Check how much time you spent in sedentary (sitting) state yesterday.	
	Feedback on behavior	Congratulations! You achieved your daily sedentary behaviour goal *[<VALUE hours]* today. Keep it up!	
**Move More**			
	Behavior substitution	It’s a beautiful day today, instead of sitting why not grab your lunch, go out and find a place to enjoy your food.	
	Social support	Invite a colleague to join you for lunch today! You can find a nice place outside to sit and enjoy!	
	Problem-solving or barrier identification	If most of your colleagues are inactive people and you think you have no company, you can encourage them or make new friends who are usually active. Invite someone to an afternoon walk now.	
	Instructions on how to perform the behavior	Getting out to walk in the rain is still a great way to connect and enjoy the outdoors. It’s time to take an umbrella and go out!	
	Providing information on the consequences of PA specific to the individual	If you have an active lifestyle it helps you manage your blood sugar. It’s time to do some outdoor light exercises in your neighbourhood.	
	Prompts or cues	Try now if you like: Walk around your building for a break during lunchtime.	
	Prompting generalization of a target behavior	Can you try to increase your activity time (while you do at work) also when you are at home? It’s the right time to be active now!	
	Goal setting	You can achieve your daily physical activity goal (*[>=VALUE minutes]*) by engaging in short walks in your backyard or nearby park, etc. Try it now!	
	Self-monitoring	Check how much time you spent in physical activities yesterday.	
	Feedback on behavior	Congratulations! You achieved your daily physical activity goal *[>=VALUE minutes]* today. Keep up the good work!	

### Overview of Procedures

Eligible participants who provide their consent will be provided with the iMove app installer and invited to a web-based individual orientation session through the Zoom videoconferencing platform. During the web-based meeting, participants will be assisted in installing the app and informed about how to use the iMove app and SORD device. Weight, height, and other demographic measures (age, gender, marital status, job status, and education level) will be assessed at baseline as well as after intervention.

### Data Collection and Storage

Activity sensor data (sitting, standing, and walking time) and other mobile data (location, weather, and calendar information) will be collected automatically through SORD and iMove and sent to a Deakin University secured server. Participant questionnaire data, including demographics questionnaire and system usability, will be captured digitally using Deakin secured Qualtrics surveys.

### Proximal Outcome

Time spent sitting (T_sit_), standing (T_s_), and walking (T_w_) within 1 hour after sending the push notification are the proximal outcomes.

### Distal Outcome

The distal outcomes of interest are the “average weekly time spent sanding and walking altogether, that is, nonsitting time” and the “average time spent being in the sitting state.” Moreover, we will also assess total sitting, standing, walking, and exercising time.

### Contextual Measures

Contextual factors, including weather and location, will be assessed using embedded smartphone sensors (GPS) and weather application programming interfaces (APIs; eg, OpenWeatherMap).

### Other Outcome Measures

Other outcome measures include feasibility and acceptability. Feasibility will be assessed with descriptive statistics, including the proportion of missing data and engagement with the app (the average time the app was opened every day). Usability is assessed using the System Usability Scale through a web-based survey at the end of the intervention [25,26].

Noncompliance with the intervention platform (ie, SORD and iMove) will be evaluated by sending reminders and phone calls. In case no data have been gathered at one decision point, an automated text message reminder will be sent to the user. This reminder will be triggered once a day. If the data remain uncollected for the second day, the system will notify the research team, and a human investigator will contact the user to encourage them to use the platform and offer assistance in resolving any problems. Each user will be expected to continue the study until they provide data for the corresponding number of days × decision times (42 days × 5 = 210).

### Data Analysis

#### Primary Analysis

Data from the 22 participants who generated 42 days of expected data, totaling 4620 decision points, will be analyzed. The study days will be continued until we reach the required number of days of data for each individual. Moreover, to deal with missing data at various decision points that could result from Bluetooth issues with the transfer of data or any other reasons (eg, activity states not being recorded), we will impute 0 for all missing minutes of sitting, standing, and walking.

MRT data will be analyzed using the weighted and centered least-squares estimator as previously described [27]. Using this analytical method, time-varying covariates can be handled, and the causal effect can be consistently estimated. Statistical analyses will be performed using R software (R Foundation for Statistical Computing) using the geepack R package.

Primary outcome measures include decision point, day, availability, interventions, and proximal change in activity states (ie, within 1 hour after the intervention is delivered). Overall effects of delivering a suggestion compared with not providing a suggestion on the subsequent 1-hour sitting, standing, and walking behaviors will be assessed. Moreover, linear treatment effects for Sit Less and Move More suggestions will evaluate the interaction between suggestion effects (ie, Sit Less or Move More) and decision points.

Statistics including β estimate, 95% CI, and *P* values will be reported for the average proximal effect as well as the multivariate effect (main effect). Sit less, Move More, and no suggestion (ie, control) conditions will be compared.

#### Secondary Analysis

Applying control systems principles [28], matchable-observable linear identification algorithms will be used to estimate linear time-invariant models from iMove intervention data [28]. Behavioral changes, including standing and walking time, will be assessed as a function of the onset, and offset of individual intervention components (ie, messages) and time-variant determinants (eg, contextual factors). Changes to the input variable (eg, behavior change technique) enable estimation and validation of computational models, therefore allowing exploration of personalized models. An individualized mathematical model of standing and walking time (outputs) will be created for Sit Less and Move More messages considering different contextual factors in different models. Also, further analysis will reveal unique intervention messages that generate the most effective behavior change (ie, either increasing standing or walking time) across various contexts.

### Ethics Approval

The study received ethical approval from the Deakin University Human Research Ethics Committee (2021-258).

## Results

The iMove app and integration with SORD were finalized in August 2022. Recruitment was initiated in October 2022. So far, 6 participants (2 female and 4 male) have provided their consent and enrolled in the study. They have completed baseline measurements and started using iMove. The mean age of 6 participants is 56.8 years, and they were diagnosed with T2D for 9.4 years on average.

## Discussion

### Overview

We present an MRT to evaluate the development of a Sit Less and Move More JITAI intervention in people with T2D [20]. The proximal and distal main effects of the sedentary and PA interventions on reducing sitting time and increasing standing or walking time (ie, nonsitting time), along with their univariate effects, will be evaluated. Moreover, the most effective behavior change technique will be recognized. Furthermore, the most critical contexts (eg, location: home or workplace) in which users are most responsive to interventions will be identified.

Too much sitting is a distinct behavior consideration from too little exercise for increasing the risk of cardiovascular disease [20]. Practical and integrated approaches involving both Sit Less and Move More have been introduced for risk reduction [20]. It is expected that interventions delivering a combination of Sit Less and Move More would add to the overall daily activity of an individual rather than focusing on at least 150 minutes of weekly exercise [20].

To our knowledge, there are no previous MRT studies targeting SB and PA among people with T2D. Other studies have focused on the general public including healthy sedentary adults [14] and university students [29]. The study by Klasnja et al [14] showed that the 6-week MRT intervention (antisedentary or walking vs no suggestion) among healthy sedentary adults (n=44) was effective to increase the average 30-minute step counts (ie, within 30 minutes after the intervention) by 14%. The study by Figueroa et al [29] conducted a 6-week MRT with university students (n=99) to improve their PA level. They showed that receiving the intervention (motivational messages and feedback on step counts vs no suggestion) increased daily steps by 729 steps, but this effect was diminished over time to –33 steps a day. They recommend that future studies need to take into account both user context and preferences [29].

These theory-based MRT studies were mainly focused on walking [14] rather than standing. Incorporating standing into the intervention package could make the Sit Less and Move More concept more practical. Replacing sitting time with standing could be considered a medium in which the most sedentary populations (eg, T2D) may make the initial transition from an inactive lifestyle to a more active lifestyle [30].

### Limitations and Strengths

Due to the data-intensive nature of this study and the reliance on mobile apps for data transfer, heavy data loads may complicate data transfer to the backend server and generate missing instances. Even though multiple troubleshooting steps have been taken to develop iMove, such issues could still occur on certain smartphones with restricted manufacturers’ system mandates. A key strength of this study is the incorporation of standing as a measured outcome. Because the focus of this MRT is on supporting people with T2D transition from sedentary to more overall physical activities, assessing transitional states, that is, standing, seems relevant. Assessing standing time, distinct from sitting and moving time, is expected to provide more robust evidence on how such Sit Less and Move More MRT performs.

### Conclusions

Overall, the results of this study will inform the optimization of digital behavior change interventions to reduce SB and increase PA among people with T2D. Moreover, the findings will provide practical implications to clinicians concerned about recommending high-intensity exercise to older people with T2D. Finally, policy makers will be informed about simple smartphone-based interventions to reduce the burden of sedentariness among people with chronic diseases (eg, diabetes).

## Appendix

Multimedia Appendix 1 An example list of iMove notification.

## Abbreviations

API: application programming interface
JITAI: just-in-time adaptive intervention
mHealth: mobile health
MRT: microrandomized trial
PA: physical activity
SB: sedentary behavior
SORD: Sedentary Behavior Detector
T2D: type 2 diabetes
WHO: World Health Organization
