# Supplementary material for: Just-In-Time Adaptive Intervention to Sit Less and Move More in People With Type 2 Diabetes: Protocol for a Microrandomized Trial
Source: JMIR Res Protoc. 2023 Sep 6;12:e41502. doi: 10.2196/41502 (PMC10512121; doi:10.2196/41502)
Supplement: Multimedia Appendix 1 [file resprot_v12i1e41502_app1.pdf]

Supplement 1. An example list of iMOVE notification.

BCT	Message	Context
Behaviour substitution (Sit less)	You have been sitting for a long time. Why not stand up to do your work instead of sitting.	Workplace
		Weekday
		Any weather
		Any time
	Why not stand up and do some leg stretches instead of spending more time seated	Any location
		Any day
		Any weather
		Any time
	Try to alternate between sitting and standing (e.g a stand-up desk) rather than sitting for all your work for the next hour.	Workplace
		Weekday
		Any weather
		Any time
	You have been sitting for about 1 hour this morning. Why not stand up and do some stretches for 2-3 minutes.	Any location
		Any day

		Any weather<br>Morning
	Have you been sitting on the couch/ chair all this weekend afternoon? Why not stand up and breathe deeply for a couple of minutes. It will energize you!	Home<br>Weekend<br>Any weather<br>Afternoo n
	Try to alternate between sitting and standing (e.g watch TV while standing for a few minutes) rather than sitting all evening.	Home<br>Any day<br>Any weather<br>Evening
	Have you been sitting on the couch/ chair all this evening? Why not stand up and stretch out your legs. This will refresh you!	Home<br>Any day<br>Any weather<br>Evening
	You've been sitting for so long, why not break your sitting and go take one of your favorite books and enjoy reading!	Home<br>Weekend<br>Any weather

		Any time
Behaviour substitution (Move more)	You’ve been sitting for long period of time why not walk around (indoor or outdoor) for a few minutes.	Any location
		Any day
		Any weather
		Any time
	Why not go out for a walk instead of spending more time seated.	Any location
		Any day
		Good weather
		Any time
	It’s a beautiful day today, instead of sitting why not grab your lunch, go out and find a place to enjoy your food.	Workplace
		Weekday
		Good weather
		Lunchtime
	You have been sitting for about 1 hour this morning. Why not go out for a 10 minutes walk while listening to music.	Any location
		Any day
		Good weather

		Morning
	Have you been sitting on the couch/ chair all this weekend afternoon? Why not swap these for some house chores (e.g. sweeping, mopping, vacuuming).	Home<br>Weekend<br>Any weather<br>Afternoon
	I know it's raining, why not grab an umbrella and go out for a short walk instead of spending more time sitting.	Any location<br>Any day<br>Bad weather (rain)<br>Any time
	You've been sitting for long period of time this evening why not walk around the house for 10 minutes.	Home<br>Any day<br>Any weather<br>Evening
	Although it's cold outside, Why not put some warm clothing on and go out for a short walk instead of spending more time sitting? You will feel the fresh air.	Any location<br>Any day<br>Cold weather

		Any time
Social support (Sit less)	Why not ask a colleague to have a coffee or tea together this afternoon!	Workplace
		Weekday
		Any weather
		Afternoon
	Ask your partner to do some house chores with you (e.g. sweeping, mopping, vacuuming, etc.). It is more fun when you do this together while talking as well!	Home
		Weekend
		Any weather
		Any time
	You've been sitting too much. Why not stand up and make a tea or coffee for your partner or friend!	Home
		Any day
		Any weather
		Any time
	You can do some gardening or any other activities you enjoy this weekend. Ask your partner to join you and have some fun!	Home
		Weekend
		Good weather
		Morning
		Home

	Sitting for a long time this weekend evening? Try calling a friend or family member but walk around while talking.	Weekend<br>Any weather<br>Evening
Social support (Move more)	What nice weather! Invite a colleague to go for a lunchtime walk.	Workplace<br>Weekday<br>Good weather<br>Lunchtime
	It's time to break up your sitting with some movement. Why not invite a friend or your partner to join you for a walk!	Home<br>Any day<br>Good weather<br>Any time
	What a weekend morning! Ask a friend or your partner to spend time (e.g. 30 to 60 minutes) walking to a nearby park.	Home<br>Weekend<br>Good weather<br>Morning
	Invite a colleague to join you for lunch today! You can find a nice place outside to sit and enjoy!	Workplace<br>Weekday

		Good weather
		Lunchtime
	Ask a friend or your partner to join you for an activity that you both like (e.g. riding a bike, walking a dog, etc.).	Home
		Weekend
		Good weather
		Any time
	Share the joy of outdoor physical activity with a friend or your partner!	Home
		Any day
		Good weather
		Any time
	Ask a coworker to take a walk while you brainstorm ideas	Workplace
		Weekday
		Good weather
		Anytime (except evening)
	This afternoon would be a good time for a challenge: walking in bad weather for 10 minutes. Invite a friend or colleague, bundle up & enjoy being active!	Any location
		Any day

		Cold weather<br>Afternoo n
Problem solving/ barrier identification (Sit less)	You might not notice your long periods of sitting time, but it adds up to a lot of hours (e.g. we may sit during meals, travelling to/from work, in front of the computer, watching TV,…). Now it's the right time to take a break and stand up and do some stretches or warm-up exercise.	Any location<br>Any day<br>Any weather<br>Any time
	Being active does not mean you have to be sporty. There's no need to overdo it. Just try to do some activity (e.g. gardening, dancing, stair climbing, stretching, etc.). Start slowly and work at your own pace. Try one activity this morning!	Home<br>Any day<br>Any weather<br>Morning
	If you can't squeeze in some outdoor exercise, you can stand up where you are, do some stretches or squats for 10 minutes! Simply be active!	Any location<br>Any day<br>Any weather<br>Any time
		Workplac e<br>Weekday

	You might not even notice you have been sitting for so long while working this afternoon. It adds up to a lot of hours! You can stand up and extend your legs and hands for a few moments and then return to work.	Any weather<br>Afternoon
Problem solving/ barrier identification (Move more)	Look around your work, is everyone sitting? You might think that you’ll be seen as an eccentric if you do some exercises. But lunchtime is a great opportunity to take your lunch and walk to a nice place to enjoy your food. This way you have done some simple exercises!	Workplace<br>Weekday<br>Good weather<br>Lunchtime
	If most of your colleagues are inactive people and you think you have no company, you can encourage them or make new friends who are usually active. Invite someone to an afternoon walk now.	Workplace<br>Weekday<br>Good weather<br>Afternoon
	If you think you don’t have enough time to exercise, the advice is that even a few minutes adds up! Select activities requiring minimal time, e.g. walking, jogging, or stair climbing. After sitting for so long, it’s time to try!	Any location<br>Any day<br>Good weather<br>Any time
		Home

	You might not even notice you have been sitting for so long. On average people spend about 8h/day sitting and 15h/day in sedentary activities if we include bedtime. Why not go for a short walk in the neighborhood.	Any day<br>Good weather<br>Any time
	If you feel like you are tired/ or not in a good mood to be physically active, you can do a little bit of indoor activity (e.g. warm-up, squats, etc.). Try it now!	Home<br>Any day<br>Any weather<br>Any time
	A rainy day might prevent you from some outdoor exercises (e.g. running, hiking, etc.), but walking in the rain is still a great way to connect and enjoy the outdoors. All you need is an umbrella and some warm clothes. Let's go for it this weekend morning.	Home<br>Weekend<br>Bad weather (rain)<br>Morning
Instruction on how to perform the behaviour (Sit less)	Time to stand up: you may want to grab a drink of water, talk on the phone while walking around or go and talk to your colleagues instead of emailing them.	Workplac e<br>Weekday<br>Any weather<br>Any time
	Get out of the chair during the TV advertisements (do little tasks, whatever you feel like).	Home<br>Any day

		Any weather<br>Any time
	Eat your lunch away from your desk. Try it today if you haven't had your lunch already.	Workplace<br>Weekday<br>Any weather<br>Lunchtime
	A weekend at home: put on some headphones and listen to music while you do house chores (e.g. sweeping, mopping, vacuuming, doing laundry, etc.).	Home<br>Weekend<br>Any weather<br>Morning/afternoon
	Stand up, do some stretches or leg squats for a couple of minutes!	Any location<br>Any day<br>Any weather<br>Any time
	Watching TV seated on the couch? Break this up by reading your book.	Home<br>Any day

		Any weather<br>Any time
	Stand up, make yourself a cup of coffee or tea and enjoy your weekend afternoon!	Home<br>Weekend<br>Any weather<br>Afternoon
	Sitting and watching TV, looking at your phone screen, or have been inactive? Give yourself a break and walk around the house for a few minutes!	Home<br>Any day<br>Any weather<br>Any time
Instruction on how to perform the behaviour (Move more)	Need to contact a colleague: Wall to your colleague instead of emailing or calling.	Workplace<br>Weekday<br>Any weather<br>Anytime
	This afternoon take the stairs instead of the lift/escalator. Or get off a few floors early and take the stairs the rest of the way.	Workplace<br>Weekday

		Any weather
		Afternoon
	Walk around your building for an active break. Time to break up your sitting time!	Workplace
		Weekday
		Any weather
		Any time
	Nice weekend, isn't it? Work in the garden, mow the grass, rake leaves, prune, dig, pick up trash, or do anything you want.	Home
		Weekend
		Good weather
		Morning/afternoon
	When you are leaving work, use the stairs instead of the elevator/escalator (IF 1 hour before leaving workplace: [usual time])	Workplace
		Weekday
		Any weather
		Afternoon
		Home

	Sitting for so long? Why not go to a nearby park and do some light exercises/ stretches.	Any day<br>Good weather<br>Morning/ afternoon
	Go outside, listen to your favourite music while walking/ exercising; enjoy something that motivates you.	Home<br>Any day<br>Good weather<br>Any time
	Getting out to walk in the rain is still a great way to connect and enjoy the outdoors. It's time to take an umbrella and go out!	Home<br>Any day<br>Bad weather (rain)<br>Any time
Providing information on the consequences of PA specific to the individual (Sit less)	Sitting is a very common human behaviour. Even for individuals who do 30 minutes of moderate physical activity on most days each week, there are adverse health consequences for those who sit for more than 7h/day. Time to break up! Stand up and do something.	Any location<br>Any day<br>Any weather<br>Any time
		Home

	It’s important to spend less time being sedentary. You’ll have more balance, as well as stronger muscles and bones. Now it’s a good time for a 10 minutes walk around the house.	Any day<br>Any weather<br>Any time
	Those who sit less have better overall health. Stand up and get the body moving such as extending your legs or raising your calves.	Any location<br>Any day<br>Any weather<br>Any time
	Grab your lunch, go out and find a place to enjoy your food. Interrupting your prolonged sitting have beneficial effects on your body’s metabolism.	Workplace<br>Weekday<br>Good weather<br>Lunchtime
	Sitting while working too much this afternoon? Stand up and do some shoulder stretch or extend your legs and stretch yourself higher for a few seconds. You’ll have more energy and it’s fun as well.	Workplace<br>Weekday<br>Any weather<br>Afternoon
		Home

	Sitting too much this evening, you can try walking inside home for a few minutes. Such simple activities can improve your sleep.	Any day<br>Any weather<br>Evening
	Plan a weekend morning outing that involves physical activity (hiking, backpacking, swimming, etc.). Stand up and gear up. You'll feel more relaxed, and less stressed.	Home<br>Weekend<br>Good weather<br>Morning
	Breaking up sitting with 5 minutes of standing, if done regularly, will help your body's ability to manage sugars and fats in the blood. Why not try it now!	Any location<br>Any day<br>Any weather<br>Any time
Providing information on the consequences of PA specific to the individual (Move more)	People who are inactive are more likely to develop diabetes, heart disease and complications of these diseases. Short walks every day reduces these consequences significantly. It's time to go out for a walk!	Any location<br>Any day<br>Good weather<br>Any time
		Any location<br>Any day

	Those who do even a little amount of exercise, not meeting the recommendations but doing something, lower their risk of hypertension and cardiovascular disease compared with inactive individuals. Why not go out for a 10 minutes walk.	Good weather<br>Any time
	It is important to take some time to be physically active. It helps reduce the risk of conditions like heart disease, obesity, type 2 diabetes, and cancer. Spend some time visiting a nearby park this morning.	Home<br>Any day<br>Good weather<br>Morning
	Those who accumulate at least 150 minutes/week of moderate activity (e.g., walking or easy cycling) have 31% lower risk of death, more weight loss and more energy compared with inactive individuals. Do jogging or walking exercises in this nice weekend!	Home<br>Weekend<br>Good weather<br>Anytime except evening
	If you have an active lifestyle it helps you manage your blood sugar. It’s time to do some outdoor light exercises in your neighborhood.	Home<br>Weekend<br>Good weather<br>Morning/ afternoo n
	Blimey! It’s a cold weekend morning. Try to go out for a walk in the stores/markets. It improves your health and well-being.	Home<br>Weekend

		Bad weather (cold)<br>Morning
	Time to do give yourself a break from work and do some physical activity. When you walk pick up the pace from leisurely to brisk. When such activities become regular you will have greater energy levels.	Workplace<br>Weekday<br>Good weather<br>Anytime
	Enjoy the fresh air by taking a walk around your neighborhood. You can concentrate better on your work when you are back!	Workplace<br>Weekday<br>Good weather<br>Anytime (except morning)
Prompts/ cues (Sit less)	Why not go take one of your favorite books and enjoy reading! This way you break up your sitting.	Home<br>Any day<br>Any weather<br>Any time
		Workplace

	Use the stairs instead of the elevator/escalator. As you leave the workplace today try it! (IF 1 hour before leaving workplace: [usual time])	Weekday<br>Any weather<br>Afternoon
	Here's something you could try to break up your sitting: Walk down the hall to speak with someone rather than calling/emailing, or just for a chat.	Workplace<br>Weekday<br>Any weather<br>Any time
	Try working while standing up: That's great if you have a stand-up desk.	Workplace<br>Weekday<br>Any weather<br>Any time
	A nice weekend morning! Work in the garden, mow the grass, rake leaves, prune, dig, and pick up trash.	Home<br>Weekend<br>Good weather<br>Morning
	Try to do one of your house chores (e.g. sweeping, mopping, vacuuming, ironing, etc.). This adds up to total activity	Home<br>Any day

		Any weather<br>Any time
	If you are sitting watching TV get up during the advertisements or if you are sitting and looking at a screen (like phone, tablet, etc.), get up and get some movement!	Home<br>Any day<br>Any weather<br>Any time
Prompts/ cues (Move more)	Go for a walk to the local store/market. That's a smart way to be physically active when it's raining!	Home<br>Weekend<br>Bad weather (rain)<br>Morning/ afternoon
	Need to use the toilet after lunch? Walk to the furthest available one in the workplace.	Workplace<br>Weekday<br>Any weather<br>Any time
	Try now if you like: Walk around your building for a break during lunchtime.	Home<br>Weekday

		Good weather<br>Lunchtime
	Go out for a short walk after dinner!	Home<br>Any day<br>Good weather<br>Evening
	If you are inactive and watching TV, take some breaks during commercials to walk around the house or do some housework.	Home<br>Any day<br>Any weather<br>Any time (except lunchtime)
	If you've been in the sitting state for so long and probably looking at a screen (such as phone, tablet, etc.) take some breaks to walk around the house.	Home<br>Any day<br>Any weather<br>Any time (except lunchtime)

	Go out for a walk instead of spending more time at home	Home<br>Any day<br>Good weather<br>Any time
Prompting generalization of a target behavior (Sit less)	It looks like you are doing well decreasing your sitting time during the week. Can you try to do it this weekend? Right now is the best time!	Home<br>Weekend<br>Any weather<br>Any time
	Can you try to interrupt your sitting time with some movement (while you do at work) also when you are at home? Try it right now!	Home<br>Any day<br>Any weather<br>Any time
	It looks like you are doing well decreasing your sitting time during the weekend. Can you try to do it in weekdays as you have been sitting for a long time at workplace? Exactly right now!	Workplac e<br>Weekday s<br>Any weather<br>Any time
	It looks like you are doing well reducing your sitting time during the mornings. Try to break your sitting time in the evenings as well. It's the time!	Any location

		Any day<br>Any weather<br>Evening
Prompting generalization of a target behavior (Move more)	It looks like you are doing well increasing your walking time during the week. Can you try to do it this weekend as you have spent so much time being sedentary (sitting)? Go for it now!	Home<br>Weekend<br>Good weather<br>Any time
	Can you try to increase your activity time (while you do at work) also when you are at home? It’s the right time to be active now!	Home<br>Any day<br>Any weather<br>Any time
	It looks like you are doing well increasing your walking time during the weekend. Can you try to do it in weekdays at your workplace as you have spent so much time being sedentary (sitting)? Go for it now!	Workplace<br>Weekdays<br>Good weather<br>Any time

	It looks like you are doing well engaging in walking activity during the lunchtimes. Try to walk and do some activities in the afternoons as well, whether indoor or outdoor. Try now!	Any location
		Any day
		Any weather
		Afternoon
Goal setting (Sit less)	To meet your daily sedentary behaviour goal [<VALUE hours], you must interrupt your prolonged sitting time. Stand up and do some stretches! <check your daily goal in the app dashboard>	Any location
		Any day
		Any weather
		Any time
	Your daily sedentary behaviour goal is [<VALUE hours]. While working, you can stand up and do some stretches. This helps you achieve your daily goal.	Workplace
		Weekdays
		Any weather
		Any time
	To help achieve your daily sedentary behaviour goal [<VALUE hours], it's a good time to do some activity (e.g. gardening, dancing, stair climbing, stretching, etc.).	Home
		Any day
		Any weather

		Any time
Goal Setting (Move more)	To meet your daily physical activity goal [>=VALUE minutes], you must increase your walking time. It’s the perfect time now to go for a short walk outside! <check your daily goal in the app dashboard>	Any location<br>Any day<br>Good weather<br>Any time
	Your daily physical activity goal is [>=VALUE minutes]. Short bouts of walking add up to your activity time. It's a great time to go for walk!	Any location<br>Any day<br>Good weather<br>Any Time
	You can achieve your daily physical activity goal ([>=VALUE minutes]) by engaging in short walks in your backyard or nearby park, etc. Try it now!	Home<br>Weekend<br>Good weather<br>Any time
Self monitoring (Sit less)	Check how much time you spent in sedentary (sitting) state yesterday.	Any location<br>Any day<br>Any weather<br>Any time

Self monitoring (Move more)	Check how much time you spent in physical activities yesterday.	Any location<br>Any day<br>Any weather<br>Any time
Feedback on behaviour (Sit less)	Congratulations! You achieved your daily sedentary behaviour goal [<VALUE hours] today. Keep it up!	Any location<br>Any day<br>Any weather<br>Anytime
Feedback on behaviour (Move more)	Congratulations! You achieved your daily physical activity goal [>=VALUE minutes] today. Keep up the good work!	Any location<br>Any day<br>Any weather<br>Anytime
